# A probabilistic early fault detection model for a feedback machining system with multiple types of spares

**DOI:** 10.1038/s41598-023-49073-6

**Published:** 2023-12-18

**Authors:** Mohamed Abd Allah El-Hadidy, Assem Omar Elshenawy

**Affiliations:** 1 Mathematics Department, Faculty of Science, Tanta University, Tanta, Egypt; 2 Department of Physics and Engineering Mathematics, Faculty of Engineering, Tanta University, Tanta, Egypt

**Keywords:** Mechanical engineering, Statistics, Mathematics and computing

## Abstract

This paper studies corrective and preventive maintenance to provide a quality control policy. The corrective maintenance, depending on the time, of a feedback machining system model with a finite source and standbys is presented. Moreover, the system has a known number of servers to repair the damaged units, and it contains an inspector to ensure the maintenance quality of the repaired units. The exact value of the probability of n units in the system will be obtained by using an efficient algorithm that depends on the Laplace transformation. To promote the concept of preventive maintenance, we use this probability to get the probability of early fault detection as a function of time and in the steady state. The applicability of this model is discussed for different system capacities.

## Introduction

Production quality control is one of the most important methods to increase production. This control participates in identifying the causes of poor production without wasting time resulting from the failure of one of the production lines. This failure leads to a waste in the factory’s productivity and then the orders on time. This leads to the customer losing patience and having to withdraw from the service system. This will cause a loss to the factory, and therefore a mechanism must be put in place to replace the stopped machine with a working one to ensure the continuity of production.

The process of replacing damaged spares has remarkable importance in the production process due to its great benefits in decreasing the failure rate of the production lines. It is an important type of finite source queueing system where the stopped servers can be replaced with another working one to ensure the continuity of the system's operation. Shekhar et al.^1–3^ and Jain et al.^4,5^ presented many classical models with some particular conditions to study the performance measures of the machine-repairing queueing systems. After the repairing process is done for the damaged unit (one of the stopped-working servers) by using the spares, it is replaced by another damaged one. The queueing system *M/M/C/N model* is similar to the factory, which has a known number of production lines. If we used the mechanism of replacing the spares to avoid wasting time and cost, then we would face the same model as that studied by Shortle et al.^6^. They applied the cold spares approach to the *M/M/C/N* model and compared this model with the classical *M/M/C/N* model. Gupta et al.^7,8^ and Jain et al.^9^ studied the relationships between the classical model of queueing systems with some concepts (for example, balking, feedback, and retention of reneged) and its counterpart, which is interested in using the spares (to repair the stopped servers).

There are multiple types of spares that can be studied on the feedback machining system, that is, (i) cold spares. It refers to any machine that requires manual configuration and adjustment in the event of a complete failure. It may also be an internal component that is being repaired or an external component that will be repaired in the event of a major machine failure. (ii) warm spares It is a method of redundancy that involves running a single machine in the background of an identical platform, ready for replacement. But if more than one machine is damaged, one of them will be replaced immediately, and the remaining machines will be repaired to replace them after a while.); and (iii) hot spares (Normally, in this type, it is on full standby but becomes immediately available in the event of a failure or malfunction of one of the underlying machines. When there is a problem, the system is modified to fuse the hot spares with their chassis. Furthermore, it reduces average operating backup time when a machine failure is detected and can be automatically overridden, as it is designed to automatically rebuild with little or no interruption. Jain and Upadhyaya^10^ dealt with a multiple-component machining system model that considers a known number of operating units and types of spare machines. They found the steady-state probabilities of the stopped units in the system by using the matrix recursive method and also some performance measures. Recently, Kotb and El-Ashkar^11^ studied an interesting feedback machining system with a finite source, standbys, and different types of spare machines. They considered the mechanism of repairing the stopped machine as the mechanism used to provide customer service in the *M/M/C/N* queueing system. In addition, they consider an inspector who can inspect the repair machine (repair server) to provide quality control of the system. They obtained the steady-state probabilities for cold, worm, and hot spares by using the iterative method and also some performance measures. They discussed an optimization economic model to show the effectiveness of this mechanism on the total expected cost, the total expected revenue, and the total expected profit. Also, Kotb and El-Ashkar^12^ discussed the effectiveness of using the inspection process on the service quality provided by a feedback *M/M/N* queueing system with balking and retention of reneged customers. The effectiveness of quality control under the inspection process in Kotb and El-Ashkar^12^ gave more optimal results, which are better than the results in Kumar et al.^13^. This is due to using the inspection process. More recently, El-Hadidy and Fakharany^14^ provided a new iterative algorithm to obtain the transient probability of a model, which was discussed by Kotb and El-Ashkar^12^. In^14^, the Laplace transform is used to get the exponential matrix to obtain the exact solution of a probabilistic dynamical system of differential equations. They studied the behavior of the probabilities of customers as functions of time, the performance measures, and the economic optimization of the model. In addition, they showed the suitability of this algorithm to deal with some queueing models as special cases. On the other hand, this transient probability has been studied extensively in many variations for different kinds of queueing systems, for example, Vijayashree and Janani^15^ and Suranga et al.^16^.

Different methods focused on studying the transient behavior of many queueing systems and giving the exact value of the probability in the cases of $n > 0$ customers in the system and the empty system at any time $t$. One of these methods is the numerical method. Ammar and Alharbi^17^ derived the Volterra integral equation of the second kind representation by using the probability generating function to get the transition probabilities of a two-processor heterogeneous system with different service rates and time-varying arrival. They compared the obtained results with the results based on the Runge–Kutta fourth-order method. In addition, they compared the associated numerical errors with the others obtained in Dharmaraja^18^ and Coyle and Zhang^19^.

The main contribution here is to study the corrective maintenance for the machining system by discovering the unit that is likely to be damaged from the point of view of reliability theory. This is done by studying the transient behavior of a feedback machining system with standbys and multiple types of spares *(M/M/C/N*) under the quality control process. In this model, Kotb and El-Ashkar^11^ provided the steady-state probability of units in the system and the probability of an empty system by using the iterative method. For studying the transient behavior of this model in the cases of cold, worm, and hot spares, we present efficient algorithms, where each one is based on finding the exponential matrix of the system of probability differential-difference equations via the Laplace transform. This will give the exact value of the probability of $n$ units in the system at any given time $t$. On the other hand, we believe that a preventive maintenance system is required to obtain high-quality production lines at a reasonable cost and within a specific system, as well as to confirm the required specifications in terms of quantity and product quality. Intelligent maintenance supported by advanced sensor technology has become critical to ensuring safe operation, as in Yang et al.^53^, where decisions have been taken that help control the risks arising from the operating system. Some new preventive maintenance policies are discussed by Yang et al.^54^ for a single two-phase system with the aim of increasing the resulting revenues affected by performance. The success of the preventive maintenance program depends on achieving the fewest breakdowns, the shortest replacement time of the damaged units, and the lowest repair costs, so there must be a kind of balance between corrective and preventive maintenance. Given the importance of time in the replacement process of damaged units, Wang et al.^55^ presented a new model through which the appropriate time to perform this operation is determined. Preventive maintenance contributes to preventing breakdowns and detecting them before they occur. To avoid cases of failure and deterioration of the units, Wang et al.^56^ presented a proposal to improve the replacement of a damaged unit with another healthy unit that is not compatible with it on the basis of condition, age, and spare parts inventory policy. Also, Wang et al.^57^ studied a model illustrating a condition-based preventive maintenance policy for balanced systems with identical components. There are different models to detect this damage (target), which appeared in the optimal search theory. El-Hadidy et al.^20–47^ provided different search techniques to detect the lost target on the line, the plane, and the space. They aimed to detect the target in the minimum amount of time and with the maximum detection probability. The methodology in this work is to examine the queuing model's transient behaviour, which was covered in Kotb and El-Ashkar^11^. Second, this queue was intended to be appropriate for talking about preventative maintenance for repairing machine systems. Detecting unit defects before they arise is essential for this maintenance to take place. Therefore, we should use one of the most significant models to detect the lost targets, such as El-Hadidy^42^. When taken as a whole, this offers a research technique for developing a new model to solve the preventative maintenance issue.

This paper is organized as follows: “List of symbols” provides a glossary of terms used in this work. The description and the formulation of our model are presented in "Model formulation". Depending on the Poisson process, this section presents two different probabilistic systems that set up the probability functions in a suitable form. In "Corrective maintenance for transient behaviour", we present the transient behavior of this model by applying the Laplace transformation to the probabilistic systems obtained in "Model formulation" to get the corresponding exponential matrix of the coefficients matrix for each system. Then, the exact solution to these systems will be obtained by using an associated algorithm and Maple code. A special case of cold spares has been presented in "Cold spares case". "Fault detection as a preventive maintenance method" used this solution to get the probability of detection and the mean time to detection as a function of time. In the final section, we discuss the conclusion and future work.

## Model formulation

**Figure 1:**
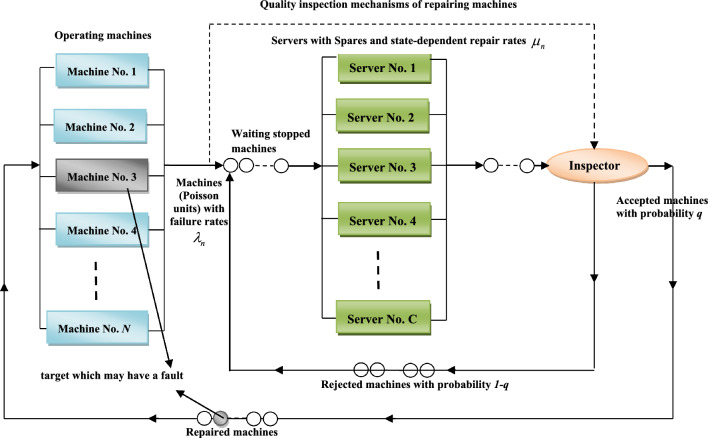
The repairing mechanism of failure machines.

Let a feedback machining system model with finite source and standbys be considered an *M/M/C/N* queueing system with spares (see Kotb and El-Ashkar^11^), where the service is provided to each unit according to the first-come, first-served (FCFS) discipline. The service and the inter-arrival time of units are independent and identically distributed (iid) exponential random variables with rates $\mu > 0$ and $\lambda > 0$, respectively, where $0 < \mu < \lambda$. The stopping machines enter the repair crews as a single waiting line, as shown in Fig. 1. The entries are done one by one according to a Poisson process.

We consider the state-dependent failure rate is given by:

1 $$\lambda_{n} = \left\{ \begin{gathered} N\lambda + S\varepsilon \begin{array}{*{20}c} {\begin{array}{*{20}c} {} \\ \end{array} } & {} & {} & {} & {} \\ \end{array} 0 \le n < S \hfill \\ (N - n + S)\lambda \begin{array}{*{20}c} {} & {\begin{array}{*{20}c} {} & {S \le n < S + N} \\ \end{array} } \\ \end{array} \hfill \\ 0\begin{array}{*{20}c} {} & {} & {} & {} & {} &\quad\quad {Otherwise} \\ \end{array} \hfill \\ \end{gathered} \right.$$

where $\varepsilon$ is a parameter that characterized the spares type and $0 < \varepsilon < \lambda$. As in Shekhar et al.^1^, if $\varepsilon = 0$ then we deal with a cold spares case. And, if $0 < \varepsilon < \lambda$ then we face a warm spares case. Otherwise, the hot spares case when $\varepsilon = \lambda$ is considered. It is clear that the behavior of the parameter $\varepsilon$ depends on the distribution of the inter-arrival time. Hence, the mechanism of repairing (servicing) depends on an identically exponential random variable with state-dependent repair rates:

2 $$\mu_{n} = \left\{ \begin{gathered} nq\mu ,\,\,\,\,\,\,\,\,\,\,\,\,\,\,\,\,\,\,\,\,\,\,\,\,\,\,\,\,\,\,\,\,\,1 \le n < C \hfill \\ \hfill \\ Cq\mu ,\,\,\,\,\,\,\,\,\,\,\,\,\,\,\,\,\,\,\,\,\,\,\,\,\,\,\,\,\,\,\,\,C \le n \le N \hfill \\ \end{gathered} \right.$$

When a malfunction occurs in one of the machines, it should be replaced by another one, and then the corrective maintenance process for the damaged machines begins. The repaired unit will become a standby for a new damaged unit. In this type of maintenance, a set of operations is provided to repair the machines through a group of servers. Maintenance times vary due to the differences in the repair process from one machine to another. After the failed machine has been repaired, it is passed on to the inspector to check its effectiveness. If the machine still fails, then the inspector will return it to the end of the original waiting line as a feedback unit with a probability of $1 - q$ for reprocessing it again (see Fig. 1). Otherwise, the not-defective machine will become a standby one to replace with another stopped or failed one on the operating system. In the case of feedback, we need to define the inspection events $\omega_{i} ,i = 1,2,...,N$ as:

3 $$\omega_{i} = \left\{ \begin{gathered} 1,\,\,\,\,\,\,\,\,\,\,\,\,\,\,\,\,\,\,\,\,\,\,\,\,\,\,\,\,\,\,\,\,\,if_{{}} the_{{}} repaired_{{}} machine_{{}} is_{{}} inspected, \hfill \\ \hfill \\ 0,\,\,\,\,\,\,\,\,\,\,\,\,\,\,\,\,\,\,\,\,\,\,\,\,\,\,\,\,\,\,\,\,if_{{}} the_{{}} repaired_{{}} machine_{{}} is_{{}} not^{{}} inspected. \hfill \\ \end{gathered} \right.$$

Here, all repaired machines are examined by the inspector to determine the quality of the repair process. For the defined policy of the inspection process, if there are $n$ jobs in the system and the repaired machine is inspected, then we have $\omega_{n} = 1$, which determines the machines that must be reconsidered or not to be repaired again; otherwise, $\omega_{n} = 0$. If the repaired machine is rejected by the inspector, then feedback must be given. This means that the existence of the feedback when the repaired machine failed to pass from the inspector is not random because it depends on the inspector's decision. When the event $\omega_{n} = 0$, this means the repaired machine has a priority to become a standby one without inspection (this may be a problem in the system where each repaired machine must be inspected before entering the standby list). By considering the above hypotheses with warm and hot spare cases (i.e., $\varepsilon \ne 0$) and applying the Markov conditions, we obtain the following basic systems of probability differential-difference equations, which have been provided in Kotb and El-Ashkar^11^. If $C \le S$, then we have a probabilistic system,

4 $$P^{\prime}_{0} (t) = - [\,\lambda \,N + S\varepsilon ]P_{0} (t) + \mu \,q\,\omega_{1} P_{1} (t)\,,\quad n = 0$$

5 $$P^{\prime}_{n} (t) = - \,[(N\lambda + S\varepsilon ) + n\mu \,q\,\omega_{n} ]P_{n} (t) + \,(n + 1)\mu \,q\,\omega_{n + 1} P_{n + 1} (t) + (N\lambda + S\varepsilon )P_{n - 1} (t),\quad 1 \le n < C$$

6 $$P^{\prime}_{n} (t) = - \,[(N\lambda + S\varepsilon ) + C\mu \,q\,\omega_{n} ]P_{n} (t) + C\mu \,q\,\omega_{n + 1} P_{n + 1} (t) + (N\lambda + S\varepsilon )P_{n - 1} (t),\quad C \le n < S$$

7 $$P^{\prime}_{n} (t) = - \,[\lambda (N - n + S) + C\mu \,q\,\omega_{n} ]P_{n} (t) + C\mu \,q\,\omega_{n + 1} P_{n + 1} (t) + (N\lambda + S\varepsilon )P_{n - 1} (t),\quad n = S$$

8 $$P^{\prime}_{n} (t) = - \,[\lambda (N - n + S) + C\mu \,q\,\omega_{n} ]P_{n} (t) + C\mu \,q\,\omega_{n + 1} P_{n + 1} (t) + \lambda (N - (n - 1) + S)P_{n - 1} (t),\;\quad S \le n \le N + S - 1$$

9 $$P^{\prime}_{n} (t) = - \,[\lambda (N - n + S) + C\mu \,q\,\omega_{n} ]P_{n} (t) + \lambda (N - (n - 1) + S)P_{n - 1} (t),\quad n = N + S$$

And, if $C > S$ then we obtain the following probabilistic system,

10 $$P^{\prime}_{0} (t) = - [\,\lambda \,N + S\varepsilon ]P_{0} (t) + \mu \,q\,\omega_{1} P_{1} (t)\,,\quad n = 0$$

11 $$P^{\prime}_{n} (t) = - \,[(N\lambda + S\varepsilon ) + n\mu \,q\,\omega_{n} ]P_{n} (t) + \,(n + 1)\mu \,q\,\omega_{n + 1} P_{n + 1} (t) + (N\lambda + S\varepsilon )P_{n - 1} (t),\quad 1 \le n < S$$

12 $$P^{\prime}_{n} (t) = - \,[\lambda (N - n + S) + n\mu \,q\,\omega_{n} ]P_{n} (t) + (n + 1)\mu \,q\,\omega_{n + 1} P_{n + 1} (t) + (N\lambda + S\varepsilon )P_{n - 1} (t),\quad n = S$$

13 $$P^{\prime}_{n} (t) = - \,[\lambda (N - n + S) + n\mu \,q\,\omega_{n} ]P_{n} (t) + (n + 1)\mu \,q\,\omega_{n + 1} P_{n + 1} (t) + \lambda (N - (n - 1) + S)P_{n - 1} (t),\quad S + 1 \le n < C$$

14 $$P^{\prime}_{n} (t) = - \,[\lambda (N - n + S) + C\mu \,q\,\omega_{n} ]P_{n} (t) + C\mu \,q\,\omega_{n + 1} P_{n + 1} (t) + \lambda (N - (n - 1) + S)P_{n - 1} (t),\quad C \le n < N + S$$

15 $$P^{\prime}_{n} (t) = - \,[\lambda (N - n + S) + C\mu \,q\,\omega_{n} ]P_{n} (t) + \lambda (N - (n - 1) + S)P_{n - 1} (t),\quad n = N + S.$$

There is a similarity between our model and the *M/M/C/N* queueing system with spares, which has been studied by Shortle et al.^6^. Our model is a generalization of the *M/M/C/N* queueing system after combining $S$ spares with it. When a machine fails in the operating system, it will be entered in the queueing model to be replaced with a spare. Then it will be entered into the inspection system. Slowness in replacing the damaged machines with proper spare parts will reduce the efficiency of the service provided. The repaired and inspected machine will become a new standby one, which will be used again in the operating system. The transient behaviour of our model will be studied in the following section to get $P_{n} (t)$ and $P_{0} (t)$ after solving the systems (4)–(9) and (10)–(15). Consequently, the performance measures of this model will be given to show its effectiveness.

## Corrective maintenance for transient behaviour

Actual corrective maintenance is a special type of maintenance activity that is undertaken to restore equipment when it has failed to meet an acceptable condition. Moreover, it is basically a correction process that is always adopted after a crash has occurred. Corrective maintenance aims to get machines back up and running as soon as possible to minimize production downtime. These goals are directly related to production capacity and costs, product quality, and consumer satisfaction. It also aims to control the investment required for backup machines. These manufacturing machines need to be replaced until the repairs are completed. As previously stated, there are three cases of spare parts replacement. To get $P_{n} (t)$, we need to solve the above systems (4)–(9) and (10)–(15). Each one can be rewritten in the following matrix form:

16 $$\dot{{\varvec{P}}}\left({\varvec{t}}\right)={\varvec{M}}{\varvec{P}}\left(t\right),$$

where if $C \le S$, then the matrix coefficients are given by, ${\varvec{M}}=({m}_{ij})\in {\mathbb{R}}^{\left(N+S+1\right)\times (N+S+1)}$ is a tri-diagonal matrix with entries given by

17 $${m}_{11}=-\left(\lambda N+S\varepsilon \right), {m}_{12}=\mu q{\omega }_{1;}$$

for $$2\le i<C+1,$$

18 $${m}_{ij}=\left\{\begin{array}{c}\lambda N+S\varepsilon , j=i-1, \\ -\left(\lambda N+S\varepsilon +(i-1)\mu q {\omega }_{i-1}\right), j=i\\ i\mu q{\omega }_{i}, j=i+1;\end{array}\right.$$

for $$C+1\le i<S+1,$$

19 $${m}_{ij}=\left\{\begin{array}{c}\lambda N+S\varepsilon , j=i-1, \\ -\left(\lambda N+S\varepsilon +C\mu q {\omega }_{i-1}\right), j=i\\ C\mu q{\omega }_{i}, j=i+1;\end{array}\right.$$

for $$i=S+1,$$

20 $${m}_{ij}=\left\{\begin{array}{c}\lambda N+S\varepsilon , j=S, \\ -\left(\lambda \left(N-i+S\right)+C\mu q {\omega }_{i-1}\right), j=S+1\\ C\mu q{\omega }_{i}, j=S+2;\end{array}\right.$$

for $$S+1<i\le N+S,$$

21 $${m}_{ij}=\left\{\begin{array}{c}\lambda (N-\left(i-1\right)+S), j=i-1, \\ -\left(\lambda \left(N-i+S\right)+C\mu q {\omega }_{i-1}\right), j=i\\ i\mu q{\omega }_{i}, j=j+1,\end{array}\right.$$

and for $$i=N+S+1,$$

22 $${m}_{i,i-1}=0, {m}_{ii}=-\left(-\lambda +C\mu q{\omega }_{N+S}\right).$$

On the other hand, the system (10)-(15) has a tri-diagonal matrix with the following entries,

23 $${m}_{11}=-\left(\lambda N+S\varepsilon \right), {m}_{12}=\mu q{\omega }_{1};$$

for $$2\le i<S+1,$$

24 $${m}_{ij}=\left\{\begin{array}{c}\lambda N+S\varepsilon , j=i-1, \\ -\left(\lambda N+S\varepsilon +(i-1)\mu q {\omega }_{i-1}\right), j=i\\ i\mu q{\omega }_{i}, j=i+1;\end{array}\right.$$

for $$S+1\le i<C+1,$$

25 $${m}_{ij}=\left\{\begin{array}{c}\lambda N+S\varepsilon , j=i-1, \\ -\left(\lambda N+S\varepsilon +C\mu q {\omega }_{i-1}\right), j=i\\ C\mu q{\omega }_{i}, j=i+1;\end{array}\right.$$

for $$i=C+1$$,

26 $${m}_{ij}=\left\{\begin{array}{c}\lambda N+S\varepsilon , j=S, \\ -\left(\lambda \left(N-i+S\right)+C\mu q {\omega }_{i-1}\right), j=S+1\\ C\mu q{\omega }_{i}, j=S+2;\end{array}\right.$$

for $$C+1<i\le N+C,$$

27 $${m}_{ij}=\left\{\begin{array}{c}\lambda (N-\left(i-1\right)+S), j=i-1, \\ -\left(\lambda \left(N-i+S\right)+C\mu q {\omega }_{i-1}\right), j=i\\ i\mu q{\omega }_{i}, j=j+1\end{array}\right.$$

and for $$i=N+C+1,$$

28 $${m}_{i,i-1}=0, {m}_{ii}=-(-\lambda +C\mu q{\omega }_{N+S})$$

The above linear system of homogenous differential equation (16), which have random coefficients, has an analytical solution with a closed form that depends on the exponential matrix (see, Hasselblatt and Katok^50^), given by

29 $${\varvec{P}}\left({\varvec{t}}\right)=exp({\varvec{M}}{\varvec{t}}){\varvec{P}}\left(0\right),$$

where ${\varvec{P}}\left(0\right)={[1 0 0\cdots 0]}^{T}\in {{\varvec{R}}}^{({\varvec{N}}+{\varvec{S}}+1)\times 1}$ is the initial condition vector. To get $exp\left({\varvec{M}}{\varvec{t}}\right),$ we apply the Laplace transformation definition (see, Chaparro and Akan^51^) on Eqs. (16) and (29). Consequently, we have

30 $$\mathcal{L}\left(exp\left({\varvec{M}}{\varvec{t}}\right)\right)={(s{\varvec{I}}-{\varvec{M}})}^{-1},$$

where $s$ is a complex variable and ${\varvec{I}}$ is the identity matrix. After that, we apply the inverse Laplace transformation (see, Dyke^52^) on Eq. (30) to get $exp\left({\varvec{M}}{\varvec{t}}\right)$. This will give the exact value of $P_{n} (t),$ and the calculation of it will be summarized in the following Algorithm 1.

### Algorithm 1

**Step 1:** Input the values of $$\lambda$$,$$\mu$$ where $$0 < \lambda < \mu$$ and $$N,$$$$C,$$$$\varepsilon$$ and $$S$$.
**Step 2:** Use the command *rand*(*0.0..1.0*) to generate the values of the probability $q$ and the command *rand*(*0..1*) to generate random integer values of $\omega_{n}$; 0 or 1.
**Step 3:** Generate a tri-diagonal matrix ${\varvec{M}}$ which contains the entries ${m}_{ij}$ given from (17)–(22) when $C \le S$ (or (23)–(28) when $C > S$).
**Step 4:** Use Laplace transform and its inverse to compute the value of (30) and then put the result in a new matrix ${\varvec{A}}$**.**
**Step 5:** Generate a column vector of the initial condition ${\varvec{I}}{\varvec{C}}$**.**
**Step 6:** Compute the value ${\varvec{A}}\times {\varvec{I}}{\varvec{C}}$ which give the exact solution of (16).

## Cold spares case

Spare parts are one of the most important factors in maintenance, but when they are available in excess, this raises production costs. If spare parts are not available, maintenance procedures are halted, and the system will stop. When $S = 0,$ any one of the above systems will become,

31 $$P^{\prime}_{0} (t) = - \,\lambda \,NP_{0} (t) + \mu \,q\,\omega_{1} P_{1} (t)\,,\quad n = 0$$

32 $$P^{\prime}_{n} (t) = - \,\,(\lambda (N - n) + n\mu \,q\,\omega_{n} )P_{n} (t) + (\,(n + 1)\mu \,q\,\omega_{n + 1} )P_{n + 1} (t) + \lambda (N - n + 1)P_{n - 1} (t),\quad 0 < n < C$$

33 $$P^{\prime}_{n} (t) = - \,\,[\lambda (N - n) + C\mu \,q\,\omega_{n} ]P_{n} (t) + \,C\mu \,q\,\omega_{n + 1} P_{n + 1} (t) + \lambda (N - n + 1)P_{n - 1} (t),\quad C \le n \le N$$

34 $$P^{\prime}_{N} (t) = - \,\,(C\mu \,q\,\omega_{N} )P_{N} (t) + \,\lambda \,P_{N - 1} (t)\,,\quad n = N$$

Similarly, one can use the above analytical method to get the exact value of $P_{n} (t)$. Example 1 discusses this solution for different system capacities.

## Fault detection as a preventive maintenance method

The main objective of preventive maintenance is to take the necessary precautions and follow the necessary procedures by discovering equipment faults to prevent accidents. Thus, to detect the fault in the repaired unit, we use the exponential detection function $1 - e^{ - z}$, where $z$ is the searching effort; see Hong et al.^48,49^. The exponential detection function provides an important feature in the process of detecting machines that will be damaged in the future because it exhibits a decreasing rate of return. This slowly increases the probability of detecting a damaged unit and increases further as the amount of searching effort increases. The probability of the unit being nominated for failure depends on the number of repaired and accepted units that have been inspected. The detection probability of the unit nominated for stopping is then given by:

35 $$P_{D} (t,n,z) = \frac{{qP_{n} (t)}}{n}\left( {1 - e^{ - z} } \right),\quad n \ge 1.$$

Now, we can compute the value of $P_{D} (t,n,z)$ for each spares case and also the mean detection time by,

36 $$E_{D} (t,n,z) = \sum\limits_{n = 1}^{N} {nP_{D} (t,n,z)} ,\quad n \ge 1$$

by adding the following two steps to the above Algorithm 1.

**Step 7.**: Use (35) to compute $P_{D} (t,n,z)$.
**Step 8.**: Use (36) to compute $E_{D} (t,n,z)$.

### Example 1

**Figure 2:**
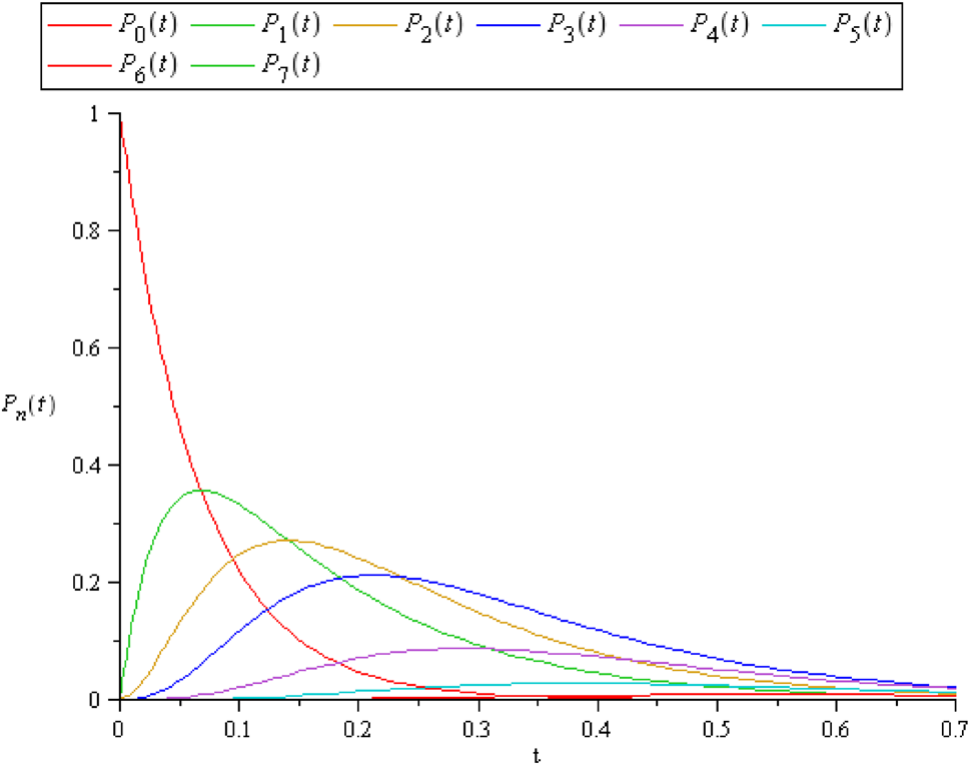
$P_{n} (t)$ when $C \le S$, where $C = 2$ and $S = 3$.

Consider the operating system contain $N = 4$ machines and different number of spared units $S$ with rate $\varepsilon = 2$. The failed units arrive to the service stage with exponential rate $\lambda = 2.3$. Also, the service time has an exponential distribution with rate $\mu = 4.72$. Thus, to get the computational value of $P_{n} (t)$, we use Maple 13 on Intel(R) Core(TM) i7 CPU with Microprocessor 2.30 GHz and with 16.0 GB. In the case of $C \le S$, where $C = 2$ and $S = 3$ the exact vector solutions $\mathbf{P}\left({\text{t}}\right)$,$t \in [0,1][0, 1]$ appears in Fig. 2, where the solution of the system (4)–(9) is obtained from the following coefficient matrices,

$$\left[ {\begin{array}{*{20}c} { - 15.2} & 0 & 0 & 0 & 0 & 0 & 0 & 0 \\ {15.2} & { - 18.05220698} & {5.704413957} & 0 & 0 & 0 & 0 & 0 \\ 0 & {15.2} & { - 15.2} & 0 & 0 & 0 & 0 & 0 \\ 0 & 0 & {15.2} & { - 18.60441369} & {5.704413957} & 0 & 0 & 0 \\ 0 & 0 & 0 & {6.9} & { - 16.30441396} & {5.70441395} & 0 & 0 \\ 0 & 0 & 0 & 0 & {4.6} & { - 14.00441369} & {5.70441395} & 0 \\ 0 & 0 & 0 & 0 & 0 & {2.3} & { - 6} & 0 \\ 0 & 0 & 0 & 0 & 0 & 0 & 0 & { - 9.404413957} \\ \end{array} } \right]$$

**Figure 3:**
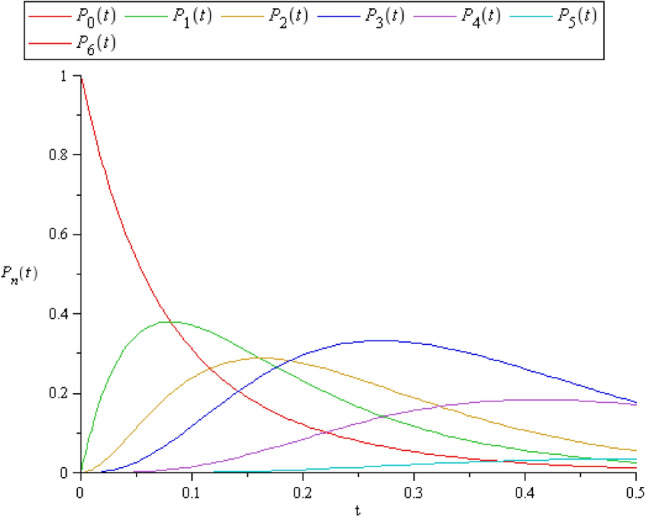
$P_{n} (t)$ when $C > S$, where $C = 3$ and $S = 2$.

Also, in the case of $C > S,$ where $C = 3$ and $S = 2$ the solution of the system (10)-(15) is obtained from the coefficient matrices to get the following $\mathbf{P}\left({\text{t}}\right),$ see Fig. 3.

$$\left[ {\begin{array}{*{20}c} { - 13.2} & 0 & 0 & 0 & 0 & 0 & 0 \\ {13.2} & { - 16.05220698} & {5.704413957} & 0 & 0 & 0 & 0 \\ 0 & {13.2} & { - 13.2} & 0 & 0 & 0 & 0 \\ 0 & 0 & {13.2} & { - 17.15662094} & {8.556620935} & 0 & 0 \\ 0 & 0 & 0 & {4.6} & { - 14.85662094} & {8.556620935} & 0 \\ 0 & 0 & 0 & 0 & {2.3} & { - 12.55662094} & {8.556620935} \\ 0 & 0 & 0 & 0 & 0 & {} & { - 1.7} \\ \end{array} } \right]$$

**Figure 4:**
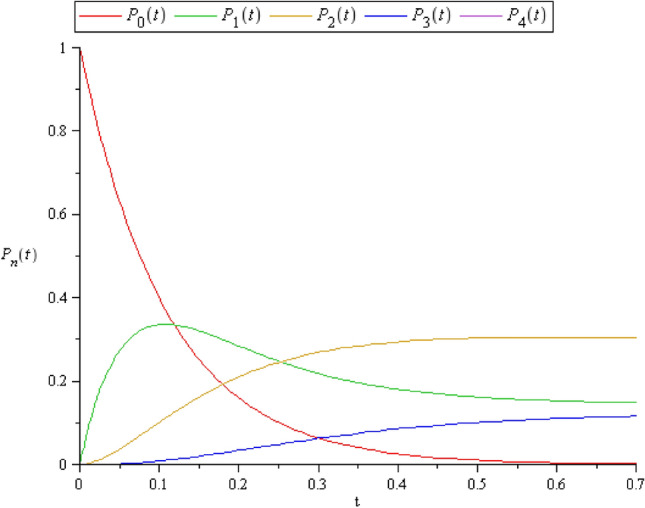
$P_{n} (t)$ in the cold spare case where $C = 3$*.*

In addition, Fig. 4 shows the exact value of $\mathbf{P}\left({\text{t}}\right)$ in the cold spare case, where the coefficient matrices given by,

$$\left[ {\begin{array}{*{20}c} { - 9.2} & 0 & 0 & 0 & 0 \\ {9.2} & { - 12.05220698} & {5.704413957} & 0 & 0 \\ 0 & {4.6} & { - 2.3} & 0 & 0 \\ 0 & 0 & {2.3} & { - 5.704413957} & {5.704413957} \\ 0 & 0 & 0 & 0 & { - 3.404413957} \\ \end{array} } \right]$$

**Figure 5:**
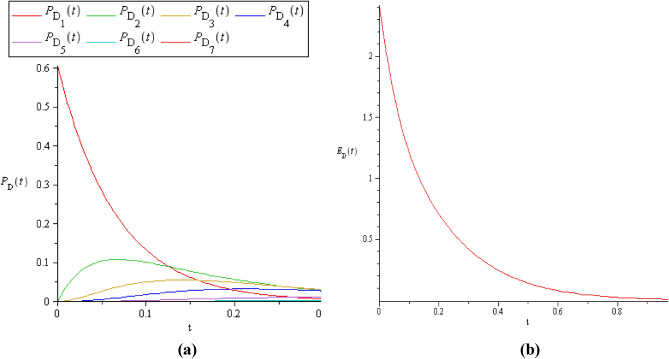
**(a)** $P_{D} (t)$ and **(b)** $E_{D} (t)$ when $C \le S$, where $C = 2$ and $S = 3$.

**Figure 6:**
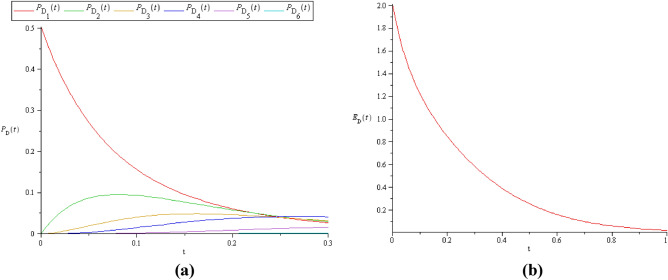
**(a)** $P_{D} (t)$ and **(b)** $E_{D} (t)$ when $C > S$, where $C = 3$ and $S = 2$.

**Figure 7:**
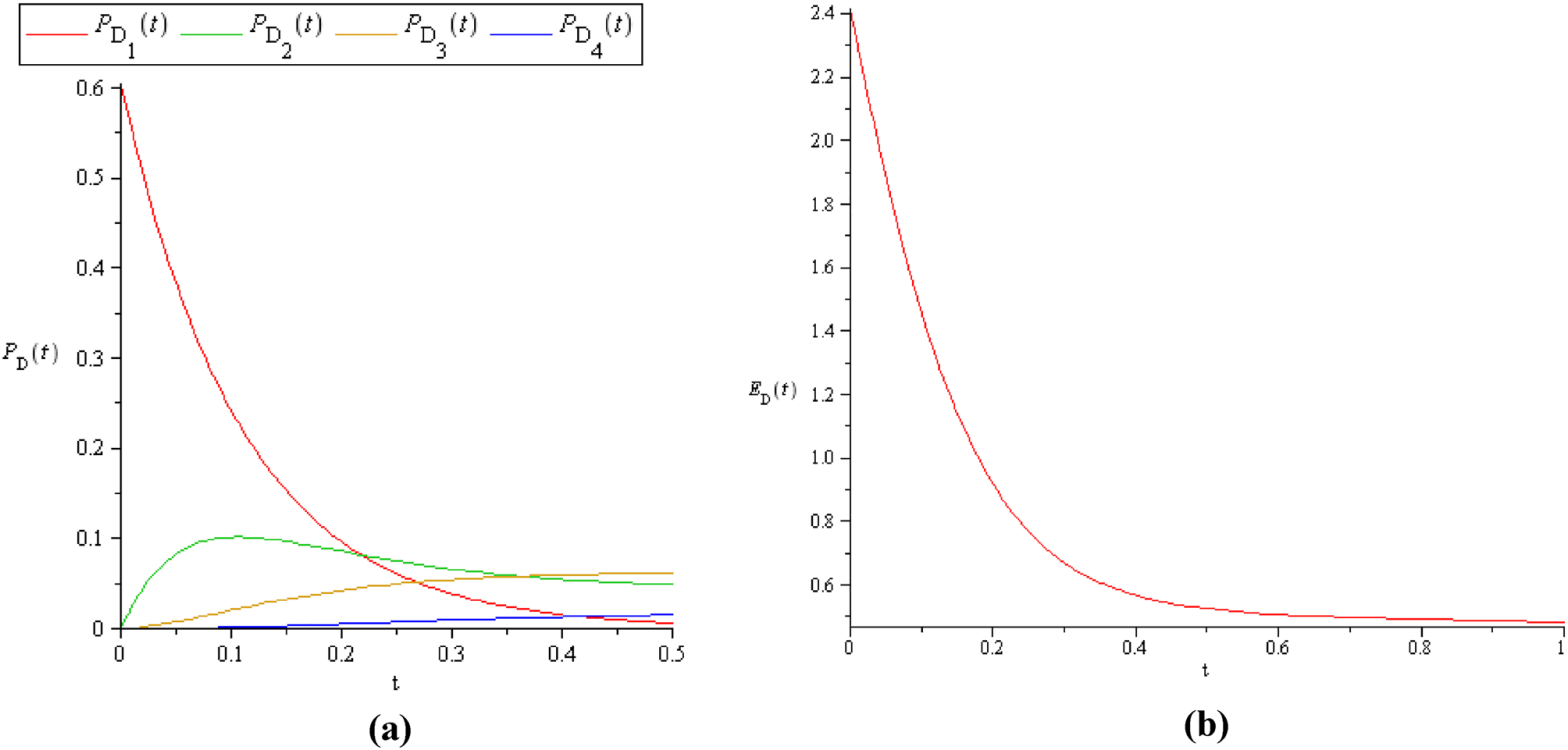
**(a)** $P_{D} (t)$ and **(b)** $E_{D} (t)$ in the cold spare case where $C = S = 2$*.*

If we consider the searching effort which used to do the early detection, is $z = 20$, then we apply the Steps 7 and 8 to get $P_{D} (t,n,z)$ and $E_{D} (t,n,z)$ for each replacement cases as in Figs. 5, 6 and 7 respectively.

It is clear that, when $C \le S$, the probability of detection will attain its maximum value (see Fig. 5a, b) because there are a greater number of standby machines than the server's. Also, this will do some maximization on $E_{D} (t)$, as in Figs. 5b, 7b.

### Steady state case

In the case of $C \le S$, the steady-state probability difference equations of the system (4)-(9) will becomes:

37 $$- [\,\lambda \,N + S\varepsilon ]P_{0} + \mu \,q\,\omega_{1} P_{1} = 0\,,\quad n = 0$$

38 $$- \,[(N\lambda + S\varepsilon ) + n\mu \,q\,\omega_{n} ]P_{n} + \,(n + 1)\mu \,q\,\omega_{n + 1} P_{n + 1} + (N\lambda + S\varepsilon )P_{n - 1} = 0,\quad 1 \le n < C$$

39 $$- \,[(N\lambda + S\varepsilon ) + c\mu \,q\,\omega_{n} ]P_{n} + C\mu \,q\,\omega_{n + 1} P_{n + 1} + (N\lambda + S\varepsilon )P_{n - 1} = 0,\quad C \le n < S$$

40 $$- \,[\lambda (N - n + S) + C\mu \,q\,\omega_{n} ]P_{n} + C\mu \,q\,\omega_{n + 1} P_{n + 1} + (N\lambda + S\varepsilon )P_{n - 1} = 0,\quad n = S$$

41 $$- \,[\lambda (N - n + S) + C\mu \,q\,\omega_{n} ]P_{n} + C\mu \,q\,\omega_{n + 1} P_{n + 1} + \lambda (N - (n - 1) + S)P_{n - 1} = 0,\quad S < n < N + S.$$

42 $$- \,[\lambda (N - n + S) + C\mu \,q\,\omega_{n} ]P_{n} + \lambda (N - (n - 1) + S)P_{n - 1} = 0,\quad n = N + S$$

and, for $C > S$, the system (10)-(15) will becomes:

43 $$- [\,\lambda \,N + S\varepsilon ]P_{0} + \mu \,q\,\omega_{1} P_{1} = 0\,,\quad n = 0$$

44 $$- \,[(N\lambda + S\varepsilon ) + n\mu \,q\,\omega_{n} ]P_{n} + \,(n + 1)\mu \,q\,\omega_{n + 1} P_{n + 1} + (N\lambda + S\varepsilon )P_{n - 1} = 0,\quad 1 \le n < S$$

45 $$- \,[\lambda (N - n + S) + n\mu \,q\,\omega_{n} ]P_{n} + (n + 1)\mu \,q\,\omega_{n + 1} P_{n + 1} + (N\lambda + S\varepsilon )P_{n - 1} = 0,\quad n = S$$

46 $$- \,[\lambda (N - n + S) + n\mu \,q\,\omega_{n} ]P_{n} + (n + 1)\mu \,q\,\omega_{n + 1} P_{n + 1} + \lambda (N - (n - 1) + S)P_{n - 1} = 0,\quad S < n < C$$

47 $$- \,[\lambda (N - n + S) + C\mu \,q\,\omega_{n} ]P_{n} + C\mu \,q\,\omega_{n + 1} P_{n + 1} + \lambda (N - (n - 1) + S)P_{n - 1} = 0,\quad C \le n < N + S.$$

48 $$- \,[\lambda (N - n + S) + C\mu \,q\,\omega_{n} ]P_{n} + \lambda (N - (n - 1) + S)P_{n - 1} = 0,\quad n = N + S.$$

Kotb and El-Ashkar^11^ used the iterative method to get the probability of $n$ units in the two cases. Thus, if $C \le S$ then

49 $$P_{n} = \left\{ \begin{gathered} P_{0} ,\begin{array}{*{20}c} {} & {} & {} & {} \\ \end{array} \begin{array}{*{20}c} {} & {} & {} & {\begin{array}{*{20}c} {} & {} & {} & {\begin{array}{*{20}c} {} & {\begin{array}{*{20}c} {} &\quad \quad {n = 0} \\ \end{array} } \\ \end{array} } \\ \end{array} } \\ \end{array} \hfill \\ \frac{{\left( {\lambda \,N + S\varepsilon } \right)^{n} }}{{n!\left( {\mu \,q} \right)^{n} \,\prod\limits_{i = 1}^{n} {\omega_{i} } }}P_{0} ,\begin{array}{*{20}c} {\begin{array}{*{20}c} {\begin{array}{*{20}c} {} & {} & {} \\ \end{array} } \\ \end{array} } & {} & {} & {} & {} \\ \end{array} 0 < n < C \hfill \\ \frac{{\left( {\lambda \,N + S\varepsilon } \right)^{n} }}{{C^{n - C} \,C!\left( {\mu \,q} \right)^{n} \prod\limits_{i = 1}^{n} {\omega_{i} } }}P_{0} ,\begin{array}{*{20}c} {} & {} & {} \\ \end{array} \begin{array}{*{20}c} {\begin{array}{*{20}c} {} & {} \\ \end{array} } & {\begin{array}{*{20}c} {} & {C \le n < S} \\ \end{array} } \\ \end{array} \hfill \\ \frac{{r^{n - S} \left( {\lambda \,N + S\varepsilon } \right)^{S} N!}}{{C!C^{n - C} \,\mu^{S} \,q^{n} (N - n + S)!\prod\limits_{i = 1}^{n} {\omega_{i} } }}P_{0} ,\begin{array}{*{20}c} {} & {} \\ \end{array} S \le n \le S + N \hfill \\ \end{gathered} \right.$$

where,

50 $$P_{0} = \left[ {\sum\limits_{n = 0}^{c - 1} {\frac{{\left( {\lambda \,N + S\varepsilon } \right)^{n} }}{{n!\left( {\mu \,q} \right)^{n} \,\prod\limits_{i = 1}^{n} {\omega_{i} } }} + \sum\limits_{n = C}^{S - 1} {\frac{{\left( {\lambda \,N + S\varepsilon } \right)^{n} }}{{C^{n - C} \,C!\left( {\mu \,q} \right)^{n} \prod\limits_{i = 1}^{n} {\omega_{i} } }} + \sum\limits_{n = S}^{N + S} {\frac{{r^{n - S} \left( {\lambda \,N + S\varepsilon } \right)^{S} N!}}{{C!C^{n - C} \,\mu^{S} \,q^{n} (N - n + S)!\prod\limits_{i = 1}^{n} {\omega_{i} } }}} } } } \right]^{ - 1} .$$

Also, if $C > S$ then we have

51 $$P_{n} = \left\{ \begin{gathered} P_{0} ,\begin{array}{*{20}c} {} & {} & {} & {} \\ \end{array} \begin{array}{*{20}c} {} & {} & {} & {\begin{array}{*{20}c} {} & {} & {} & {\begin{array}{*{20}c} {} & {\begin{array}{*{20}c} {} &\quad \quad {n = 0} \\ \end{array} } \\ \end{array} } \\ \end{array} } \\ \end{array} \hfill \\ \frac{{\left( {\lambda \,N + S\varepsilon } \right)^{n} }}{{n!\left( {\mu \,q} \right)^{n} \,\prod\limits_{i = 1}^{n} {\omega_{i} } }}P_{0} ,\begin{array}{*{20}c} {\begin{array}{*{20}c} {\begin{array}{*{20}c} {} & {} & {} \\ \end{array} } \\ \end{array} } & {} & {} & {} & {} \\ \end{array} 0 < n \le S \hfill \\ \frac{{r^{n - S} \left( {\lambda \,N + S\varepsilon } \right)^{S} N!}}{{n!\left( {\mu \,q} \right)^{S} (N - n + S)!\prod\limits_{i = 1}^{n} {\omega_{i} } }}P_{0} ,\begin{array}{*{20}c} {\begin{array}{*{20}c} {} & {} \\ \end{array} } & {\begin{array}{*{20}c} {} & {S + 1 \le n < C} \\ \end{array} } \\ \end{array} \hfill \\ \frac{{r^{n - S} \left( {\lambda \,N + S\varepsilon } \right)^{S} N!}}{{C^{n - C} \,C!\mu^{S} \,q^{n} (N - n + S)!\prod\limits_{i = 1}^{n} {\omega_{i} } }}P_{0} ,\begin{array}{*{20}c} {} & {} \\ \end{array} C \le n \le S + N \hfill \\ \end{gathered} \right.$$

where,

52 $$P_{0} = \,\left[ {\sum\limits_{n = 0}^{S} {\frac{{\left( {\lambda \,N + S\varepsilon } \right)^{n} }}{{n!\left( {\mu \,q} \right)^{n} \,\prod\limits_{i = 1}^{n} {\omega_{i} } }} + \sum\limits_{n = S + 1}^{C - 1} {\frac{{r^{n - S} \left( {\lambda \,N + S\varepsilon } \right)^{S} N!}}{{n!\left( {\mu \,q} \right)^{S} (N - n + S)!\prod\limits_{i = 1}^{n} {\omega_{i} } }}} } + \sum\limits_{n = C}^{N + S} {\frac{{r^{n - S} \left( {\lambda \,N + S\varepsilon } \right)^{S} N!}}{{C^{n - C} \,c!\mu^{S} \,q^{n} (N - n + S)!\prod\limits_{i = 1}^{n} {\omega_{i} } }}} } \right]^{ - 1} .$$

Hence, the detection probability of the unit nominated for stopping becomes,

53 $$P_{D} (n,z) = \frac{{qP_{n} }}{n}\left( {1 - e^{ - z} } \right),\quad n \ge 1$$

Also, the mean detection time becomes,

54 $$E_{D} (n,z) = \sum\limits_{n = 1}^{N} {nP_{D} } (n,z),\quad n \ge 1$$

In the cold spars case, the steady-state probability difference equations of the system (31)-(34) will becomes:

55 $$- \lambda \,NP_{0} + \mu \,q\,\omega_{1} P_{1} = 0\,,\quad n = 0$$

56 $$- \,\,(\lambda (N - n) + n\mu \,q\,\omega_{n} )P_{n} + \,\lambda \,(N - n + 1))P_{n - 1} + ((n + 1)\mu \,q\,\omega_{n + 1} )P_{n + 1} = 0\,,\quad 0 < n < C$$

57 $$- \,\,(\lambda (N - n) + C\mu \,q\,\omega_{n} )P_{n} + \,\lambda \,(N - n + 1)P_{n - 1} + C\mu \,q\,\omega_{n + 1} P_{n + 1} = 0\,,\quad c \le n \le N$$

58 $$- \,c\mu \,q\,\omega_{N} P_{N} + \,\lambda \,P_{N - 1} = 0\,,\quad n = N$$

Using the iterative method, we get

59 $$P_{n} = \left\{ \begin{gathered} \frac{{r^{n} \,}}{{q^{n} \,\prod\limits_{i = 1}^{n} {\omega_{i} } }}\left( \begin{gathered} N \hfill \\ n \hfill \\ \end{gathered} \right)P_{0} ,\begin{array}{*{20}c} {} & {\begin{array}{*{20}c} {} & {} & {} \\ \end{array} } & {1 \le n < C} \\ \end{array} \hfill \\ \frac{{r^{n} n!}}{{\prod\limits_{j = 1}^{n} {\omega_{j} } \,c!\,q^{n} C^{n - C} }}\left( \begin{gathered} N \hfill \\ n \hfill \\ \end{gathered} \right)P_{0} ,\begin{array}{*{20}c} {} & {} & {C \le n \le N} \\ \end{array} \hfill \\ \end{gathered} \right.$$

where,

60 $$P_{0} = \,\left[ {\sum\limits_{n = 0}^{C - 1} {\left( \begin{gathered} N \hfill \\ n \hfill \\ \end{gathered} \right)\frac{{\Omega^{n} \,}}{{\,\prod\limits_{i = 1}^{n} {\omega_{i} } }} + \frac{{C^{C} }}{C!}\sum\limits_{n = C}^{N} {\frac{{K^{n} n!\,}}{{\left( {N - n} \right)\,!\prod\limits_{j = 1}^{N} {\omega_{j} } }}} } } \right]^{ - 1} .$$

One can use (53) and (54) to get $P_{D} (n,z)$ and $E_{D} (n,z)$, respectively.

#### Example 2

**Figure 8:**
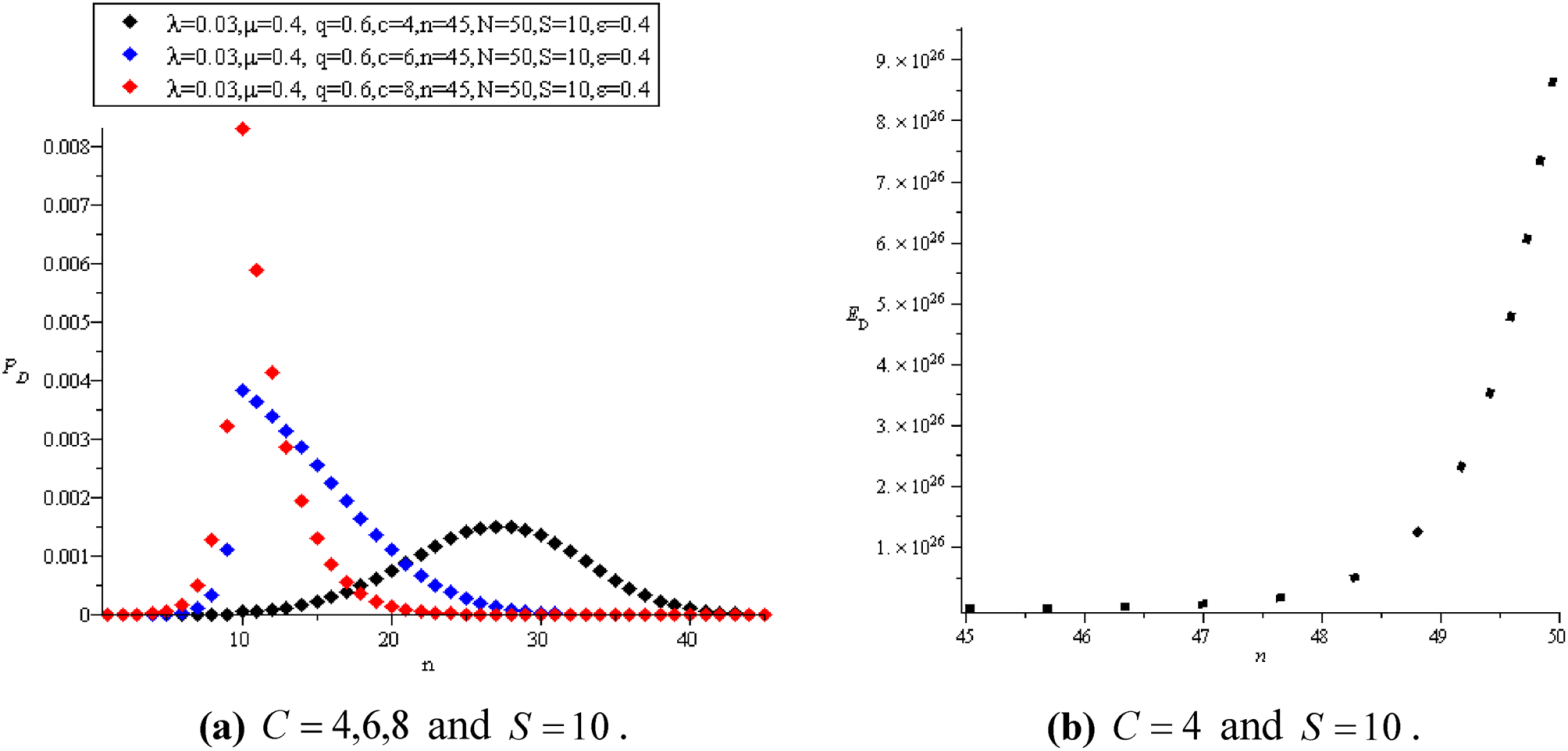
**(a)** $$P_{D}$$ and **(b)** $$E_{D}$$ when $$C \le S$$.

The main purpose of maintenance is to help the project achieve the goals for which it was established. The primary responsibility is to maximize the percentage of time the machines and equipment are available for operation. Also, maintain the value of the factory by decreasing the rates of machinery wear and deteriorating performance as a result of operation. It is known that good maintenance planning depends on estimating the cost of realistic repairs. Therefore, deviation from the estimated maintenance costs upsets the production costs and causes the estimated budget to be depleted. Therefore, the continuous detection of defects in the machines ensures their accurate work and reduces losses. If we have $N = 50$ machines and spared units $S = 10$ with rate $\varepsilon = 0.4$ such that $\lambda = 0.03$ and $\mu = 0.4$,$z = 20$ then in the case of $C = 4,6,8$ (i.e.,$C \le S$) we use (53) and (54) to get the computational values of $P_{D} (n,z)$ and $E_{D} (n,z)$ as in Fig. 8.

**Figure 9:**
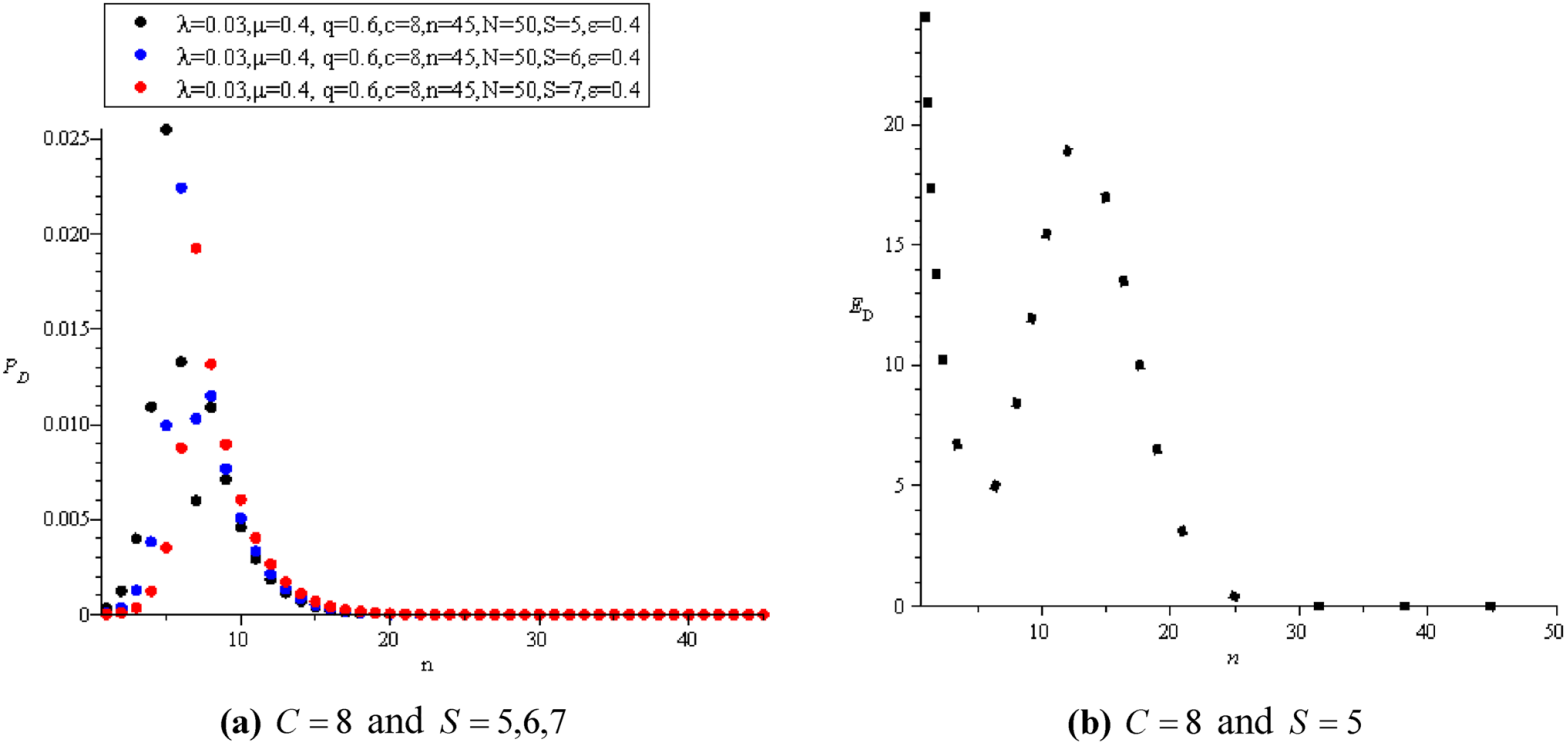
**(a)** $$P_{D}$$ and **(b)** $$E_{D}$$ when $$C > S$$.

Also, in the case of $C > S,$ where $C = 8$ and $S = 5,6,7$ the values of $P_{D} (n,z)$ and $E_{D} (n,z)$ appear in Fig. 9.

**Figure 10:**
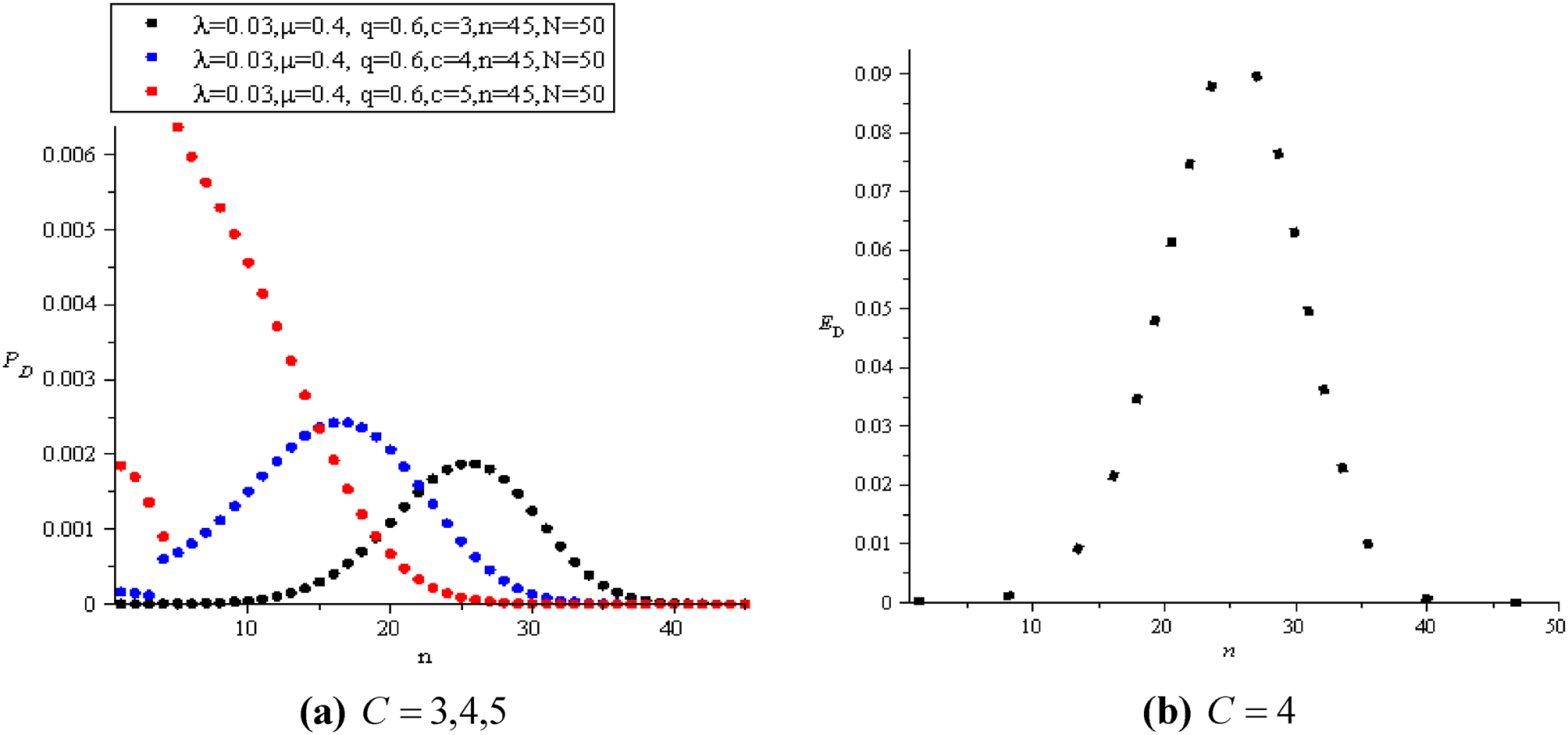
**(a)** $P_{D}$ and **(b)** $E_{D}$ in the cold spare case.

Figure 10 shows the values of $P_{D} (n,z)$ and $E_{D} (n,z)$ in the cold spare case.

When the value of spares increases and there is some stability in all other parameters, we notice a stable and noticeable increase in the value of $P_{D}$, as in Fig. 10a. In contrast to the increase in the number of servers and the stability of the rest of the parameters, a turbulent increase was observed in $P_{D}$, as shown in Fig. 9a. This is due to the change in the number of units exiting after the inspection process. A disturbance in the detection probability led to a reduction in the time to detect the fault. Of course, this had a significant impact on $E_{D}$, as shown in Figs. 9b, 10b.

## Conclusion and future work

A probabilistic model is presented for the maintenance process of $N$ machines (system capacity) in a specific operating system. This process was divided into two types of maintenance: one corrective and the other preventive. The corrective maintenance process is carried out on a group of machines that are actually broken and are waiting in a queue. There are a limited number of servers responsible for the repair process, with an inspector to ensure the quality of the service. At the same time, there are units (spares or standby) ready for replacement in the event of any malfunction. We studied the replacement process for three types of spares, which are warm, hot, and cold spares. We discussed the transient behaviour of this probabilistic model using the Laplace transform. An algorithm for calculating the exact value of the probability $n$ units ($n \le N$) over time is presented. On the other hand, preventive maintenance was studied through the early detection process of faulty units in order to avoid a long interruption of production as a result of the unit replacement process. The probability of early detection of the target and the expected value of the detection time were obtained. In addition, the detection probability and the mean time of detection are discussed in the steady-state case.

In future work, this model can be used to study preventative maintenance when the amount of the detection effort is a random variable with a known distribution. Furthermore, the flexibility of this model allows us to investigate more complex problems in real life and contains more conditions that are processed in Poisson equations.

## Data Availability

The datasets used and/or analysed during the current study are available from the corresponding author on reasonable request.

## List of symbols

*P*_*n*_(*t*): Probability of $n$ stopped machines (units) in the system at time $t$
*P*_0_(*t*): Probability of non stopping units in the system at time $t$
*n*: The number of stopped units, $0 \le n \le N$
*N*: The system capacity (all machines in the system)
*C*: The servers-technical staff (who replace damaged parts)
*S*: Spares (standby machines)
*q*: The departure probability of repaired and effective machine to the operating system
*ε*: The spares rate
*P*_*D*_(*t, n, z*): Detection probability of another further damage in the future
*E*_*D*_(*t, n, z*): Mean detection time
